# TLR4 Promotes and DAP12 Limits Obesity‐Induced Osteoarthritis in Aged Female Mice

**DOI:** 10.1002/jbm4.10079

**Published:** 2018-10-04

**Authors:** Evangelia Kalaitzoglou, Erika Barboza Prado Lopes, Yao Fu, Jacquelyn C Herron, Josiah M Flaming, Elise L Donovan, Yanqing Hu, Adrian Filiberti, Timothy M Griffin, Mary Beth Humphrey

**Affiliations:** ^1^ University of Kentucky Barnstable Brown Diabetes Center Department of Pediatrics University of Kentucky College of Medicine Lexington KY USA; ^2^ Oklahoma Medical Research Foundation Aging and Metabolism Research Program Oklahoma City OK USA; ^3^ University of Oklahoma Health Sciences Center Department of Medicine Oklahoma City OK USA; ^4^ Oklahoma City Veteran's Affairs Medical Center Department of Medicine Oklahoma City OK USA

**Keywords:** OSTEOARTHRITIS, AGING, OSTEOIMMUNOLOGY, BONE–FAT INTERACTIONS, GENETIC ANIMAL MODELS

## Abstract

Aging and female sex are the strongest risk factors for nontraumatic osteoarthritis (OA); whereas obesity is a modifiable risk factor accelerating OA. Prior studies indicate that the innate immune receptor toll‐like receptor 4 (TLR4) mediates obesity‐induced metabolic inflammation and cartilage catabolism via recognition of damage‐associated molecular patterns and is increased with aging in OA joints. TLR4 responses are limited by innate immunoreceptor adapter protein DNAX‐activating protein of 12kDA (DAP12). We undertook this study to test the hypothesis that TLR4 promotes, whereas DAP12 limits, obesity‐accelerated OA in aged female mice. We fed 13‐ to 15‐month‐old female WT, TLR4 KO, and DAP12 KO mice a high‐fat diet (HFD) or a control diet for 12 weeks, and changes in body composition, glucose tolerance, serum cytokines, and insulin levels were compared. Knee OA was evaluated by histopathology and μCT. Infrapatellar fat pads (IFPs) were analyzed by histomorphometry and F4/80+ crown‐like structures were quantified. IFPs and synovium gene expression were analyzed using a targeted insulin resistance and inflammation array. All HFD‐treated mice became obese, but only WT and TLR4 KO mice developed glucose intolerance. HFD induced cartilage catabolism in WT and DAP12 KO female mice, but not in TLR4 KO mice. Gene‐expression analysis of IFPs and synovium showed significant differences in insulin signaling, adipokines, and inflammation between genotypes and diets. Unlike young mice, systemic inflammation was not induced by HFD in the older female mice independent of genotype. Our findings support the conclusion that TLR4 promotes and DAP12 limits HFD‐induced cartilage catabolism in middle‐aged female mice. © 2018 The Authors *JBMR Plus* published by Wiley Periodicals, Inc. on behalf of American Society for Bone and Mineral Research.

## Introduction

Osteoarthritis (OA) is the most prevalent form of arthritis, affecting millions of people worldwide. Many factors contribute to the development and progression of OA, including age, injury, sex, and obesity.^1^ Obesity is the most significant preventable risk factor for OA; notably, it increases the lifetime risk of knee OA comparable to that of previous knee trauma.^2^ One consequence of the increased risk of knee OA caused by obesity is that the onset of disease occurs at a younger age. Using data from the National Health Interview Survey, Losina and colleagues estimated that obesity accelerated the onset of symptomatic knee OA by ≥15 years in women and ≥10 years in men compared with 65‐year‐old nonobese persons.^3^ For mice, this is roughly equivalent to an accelerated onset of OA by 3 to 4 months compared with that observed in a 20‐month‐old animal. In general, when OA is initiated in mice via high‐fat diet‐ (HFD‐) induced obesity without an additional injury, diet durations of 9 to 12 months are required to increase the disease severity.^4^, ^5^ Interestingly, a small reduction in body weight (5 kg), and in particular body fat (2.4%), slows the progression of OA, supporting a role for metabolic factors.^6^, ^7^ The molecular pathways directly linking obesity‐induced systemic or local joint inflammation to cartilage catabolism and OA progression are still unknown, but the dysfunction of innate immune responses may be a link.^8^, ^9^

Toll‐like receptor 4 (TLR4), a modulator of innate immunity, contributes to insulin resistance^10^ and to OA pathogenesis.^11^ TLR4 activation induces nuclear factor kappa‐light‐chain‐enhancer of activated B cells (NF‐κB) signaling and proinflammatory cytokine production, which are both upregulated in OA joint tissues.^12^ Moreover, mice lacking TLR4 are partially protected against insulin resistance and adipose tissue inflammation caused by HFD‐induced obesity.^(13,14)^ This protective effect was specifically due to the lack of TLR4‐dependent signaling in hematopoietic‐derived cells.^15^ We and others have previously shown that TLR4‐mediated cytokine responses are inhibited by DAP12 when paired with triggering receptor expressed on myeloid cells 2 (TREM2).^16^, ^17^, ^18^, ^19^ Thus, DAP12 may regulate TLR4‐dependent inflammation occurring in the synovium, infrapatellar fat pad (IFP), cartilage, or subchondral bone associated with HFD‐induced OA.

In this study, we tested the hypothesis that TLR4 and DAP12 regulate HFD‐accelerated knee OA in aged female mice. We specifically evaluated the contribution of the IFP, synovium, and subchondral bone in our model. We selected aged female mice for this study based on the higher prevalence of knee OA in older women and the importance of age in establishing physiologic mechanisms of OA pathogenesis. By comparing HFD‐induced changes in systemic and local joint inflammation in animal models that modulate TLR4 signaling positively and negatively, we provide new insight into how TLR4‐dependent pathways are activated and regulated in obesity, aging, and OA.

## Subjects and Methods

### Mice

Female C57BL/6 (WT) and TLR4 KO mice on a C57BL/6 background were purchased from Jackson Laboratories (Bar Harbor, ME, USA) and aged on site. DAP12 KO^20^ mice on a C57BL/6 background were provided by Dr RP McEver from the Oklahoma Medical Research Foundation (OMRF; Oklahoma City, OK, USA) and were bred and aged on site. Mice were group‐housed (≤5 per cage) at the OMRF vivarium on a 14‐:10‐hour light:dark cycle and allowed ad libitum access to food and water. At 13 to 15 months of age, mice were randomly assigned to irradiated control diet (10% kcal fat) or HFD (60% kcal fat) for 12 weeks (D12450Ji and D12492i, respectively; Research Diets, New Brunswick, NJ, USA). Studies were approved by the OMRF and Oklahoma University Health Sciences Center Animal Care and Use Committees.

### Body weights, body composition, and glucose tolerance

Prior to and following the initiation of diets, body composition was evaluated using a dual‐energy X‐ray absorptiometry system (Lunar PIXImus2; GE Lunar Corp, Madison, WI, USA). Mice were weighed weekly. Glucose tolerance testing was performed as previously described, using a 6‐hour fast and glucose dosing based on lean tissue mass (2 mg/g).^21^ Contour Next glucometer and test strips (Bayer Corp, Whippany, NJ, USA) were used for glucose measurements with the area under the curve (AUC) calculated based on the trapezoidal rule.

### Serum biomarker analysis

Serum was isolated from blood collected by cardiac puncture at the time of euthanasia and aliquoted for storage at −80°C until further use. Serum was diluted 1:2 for analysis of IL‐1, TNF‐α, IL‐6, IL‐10, IL‐18, IP‐10, MCP‐1, and leptin using a custom mouse Procarta Multiplex Assay (EPX080‐25016‐801; Thermo Fisher Scientific, Waltham, MA, USA). Serum was diluted 1:2000 for adiponectin analysis using a mouse Procarta Singleplex Assay (EPX01A‐26038‐901; Thermo Fisher Scientific). Insulin was quantified by ELISA (ALPCO 80‐INSMR‐CH01; ALPCO, Salem, NH, USA). Sample values below the lowest limit of detection (LLOD) were assigned a value one‐half LLOD for statistical purposes.

### Histological and μCT analysis

The left knee was fixed in 4% paraformaldehyde and underwent high‐resolution μCT scanning throughout the knee using a vivaCT 40 scanner (Scanco Medical, Basserdorf, Switzerland). Scans were used for semiquantitative grading of tibial osteophyte formation (0 to 3 scale) by three blinded observers as previously described.^22^ Subchondral bone was analyzed for tibial cortical bone volume and tibial subchondral cancellous bone volume/tissue volume. Following μCT scanning, joints were prepared for histological evaluation as previously described.^(23)^ Magnification images (×20) were obtained using a Bioquant imaging system (Version 14.1.6; Bioquant Image Analysis Corp, Nashville, TN, USA), and an Osteoarthritis Research Society International (OARSI) score, as well as a modified Mankin scores were assigned under blinded conditions by two experienced graders throughout the medial and lateral tibia and femur.^24^ Knee sections containing the IFP were stained with hematoxylin and eosin; an average adipocyte size was quantified using Bioquant software (Version 17.2; Bioquant Image Analysis Corp). Adipose and joint tissue macrophages were quantified by immunohistochemistry with a F4/80 antibody (clone BM8; Santa Cruz Biotechnology, Santa Cruz, CA, USA) following antigen retrieval and blocking. Secondary peroxidase conjugated ImmPRESS anti‐rat‐antibody reagent kit (Vector Laboratories, Burlingame, CA, USA) was used according to the manufacturer's protocol with a secondary antibody only as a negative control. All sections were semiquantitatively scored in a blinded fashion for F4/80 positive cells. Synovial thickening was also assessed in a blinded fashion using a semiquantitative scale.

### RNA isolation/gene expression

IFP and adjacent synovial and capsular tissues were dissected from the right knee under a stereomicroscope, placed in TRIzol (Ambion, Austin, TX, USA) on ice, and then stored at −80°C until homogenization. mRNA was isolated following the manufacturer's protocol and purified using a micro‐column kit (RNA Clean‐up and Concentration; Zymo Research, Irvine, CA, USA). Gene expression was quantified using a RT^2^ Profiler PCR Array (Mouse Insulin Resistance Array; PAMM 156Z, modified to include four additional genes: *Itgax, Trem2, Tryobp*, and *Mrc1*; QIAGEN, Valencia, CA, USA). Then 200‐ng mRNA per array was synthesized into cDNA using a RT^2^ First Strand Kit (QIAGEN) following the manufacturer's protocol. Samples were analyzed on an ABI 7500 thermocycler (Applied Biosystems, Foster City, CA, USA), and gene expression was quantified relative to the geometric mean of five stable reference genes: *Actb, B2m, Gapdh, Gusb,* and *Hsp90ab1*. To evaluate the effect of the HFD, fold‐change gene expression was calculated by the ΔΔC_t_ method.

### Statistical analysis

Statistical analyses were performed using GraphPad Prism 6 (GraphPad Software, La Jolla, CA, USA). The effects of a HFD and genotype were analyzed by one‐way or two‐way ANOVA. Significant treatment effects were further evaluated using Tukey's or Fisher's LSD test, respectively, to define specific group differences. As needed, data were log‐transformed to meet the statistical model assumptions. When transformation was inadequate, or for analyses of noncontinuous data such as semiquantitative scores, nonparametric Mann–Whitney *U* tests or Kruskal–Wallis one‐way ANOVAs followed by Dunn's tests were utilized. Gene‐expression array data were adjusted for multiple comparisons using the Benjamini–Krieger two‐stage linear step‐up procedure. *P* values <0.05 were considered statistically significant. Sample sizes are given in the figure legends.

## Results

### High‐fat diet induces obesity independently of TLR4 or DAP12

**Figure 1:**
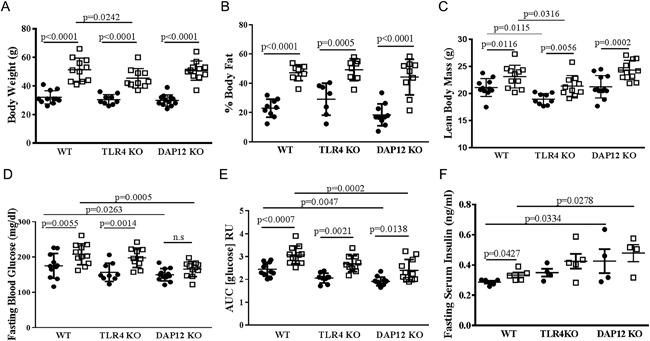
High‐fat diet induces obesity in aged female mice independent of TLR4 and DAP12. Age‐matched 13‐ to 15‐month‐old female mice, WT (*n* = 22), TLR4 KO (*n* = 19), DAP12 KO (*n* = 23), were randomized to a control diet or high‐fat diet (HFD) treatment for 12 weeks. Black circles indicate control diet and open squares represent HFD. (*A*) Body weight, (*B*) % body fat, (*C*) lean body mass, (*D*) fasting blood glucose, (*E*) 2 mg/g glucose tolerance test with blood glucose area under the curve measured out to 120 min, and (*F*) fasting serum insulin. *N* = 9 to 12 per genotype and diet groups in panels *A* through *E*; *N* = 4 to 5 per genotype and diet groups in panel *F*. Values are mean ± SD. *P* values as indicated.

We first assessed the role of TLR4 and DAP12 in regulating body mass and composition before and after HFD treatment. No significant differences in body weight, percent body fat, or lean body mass were observed between the genotypes prior to HFD treatment (data not shown). HFD treatment increased body weight, percent body fat, and lean body mass in all genotypes (Fig. 1 *A*–*C*). No significant differences in body weight or percent body fat were observed between genotypes within a given diet. TLR4 KO mice had lower lean body mass compared with WT mice, irrespective of diet (Fig. 1 *C*). Overall, 12‐weeks of HFD treatment induced significant obesity in aged female mice independent of TLR4 or DAP12 deletion.

### DAP12 KO mice are partially protected from HFD‐induced glucose intolerance

After 12 weeks of diet treatment, fasting blood glucose and glucose tolerance testing was performed. Compared with the control diet, HFD increased fasting blood glucose in WT and TLR4 KO mice, but not significantly in DAP12 KO mice (Fig. 1 *D*). Fasting blood glucose was similar between WT and TLR4 KO mice independent of diet. Compared with WT mice, DAP12 KO mice had significantly lower fasting glucose on both control and HF diets (Fig. 1 *D*). In response to a glucose challenge, the glucose area AUC was significantly lower in DAP12 KO mice compared with WT mice, independent of 12 weeks of control or HF diet (Fig. 1 *E*). TLR4 KO mice had intermediate glucose AUC between WT and DAP12 KO mice (Fig.1 *D*, *E*). Compared with WT mice, DAP12 KO mice had significantly elevated insulin levels independent of diet, and TLR4 KO mice had insulin levels intermediate between WT and DAP12 KO mice (Fig. 1 *F*). These data suggest that DAP12 and TLR4 participate in glucose homeostasis in response to HFD.

### DAP12 KO female mice are protected from age‐related knee OA

**Figure 2:**
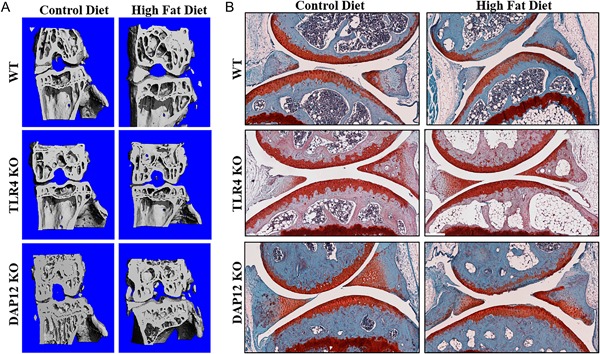
TLR4 KO mice are protected whereas DAP12 KO mice have accelerated high‐fat diet‐induced osteoarthritis progression. (*A*) Representative 3‐D reconstructed μCT images of the knees of WT, TLR4 KO, and DAP12 KO mice on control or HFD. (*B*) Representative images of medial knee joints stained with hematoxylin, safranin‐O, and fast‐green.

**Figure 3:**
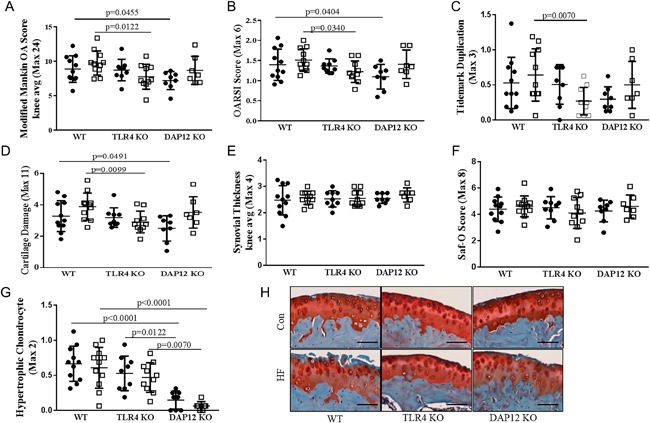
DAP12 KO mice have accelerated high‐fat (HF) diet‐induced cartilage catabolism. (*A*–*G*) Scores in panels *A* through *G* reported as the averages of four sites (medial femur, medial tibia, lateral femur, and lateral tibia). Black circles indicate a control (CON) diet and open squares represent a HF diet. (*A*) Modified‐Mankin osteoarthritis (OA), (*B*) OARSI OA, (*C*) tidemark duplication, (*D*) cartilage damage, (*E*) synovial thickening, (*F*) safranin‐O loss, and (*G*) hypertrophic chondrocytes. (*H*) Representative histology (×20) showing cartilage loss in WT and DAP12 KO mice, but preservation in TLR4 KO mice. WT CON (*n* = 11), WT HF (*n* = 11), TLR4 KO CON (*n* = 9), TLR4 KO HF (*n* = 10), DAP12 KO CON (*n* = 8), DAP12 KO HF (*n* = 7). Values are mean ± SD. *P* values as indicated.

Following 12 weeks of control or HFD treatment, knee joints were collected for μCT and histological OA analysis (Figs. 2 and 3). Control‐diet WT and TLR4 KO mice had similar low‐grade modified Mankin^23^ and OARSI OA scores (Fig. 3 *A*, *B*). Compared with WT mice, DAP12 KO mice had significantly less age‐dependent OA with reduced Mankin and OARSI scores on control diet (Figs. 2 *B*, 3 *A*, *B*). Cartilage damage and hypertrophic chondrocyte scores were significantly lower in control‐diet DAP12 KO mice compared with WT mice (Fig. 3 *D*, 3 *G*, *H*). There were no significant differences in synovial thickening or safranin‐O staining in control‐diet mice independent of genotype. These data indicate that aged female DAP12 KO mice are protected from age‐related OA.

### TLR4 KO mice are protected, whereas DAP12 KO mice have accelerated HFD‐induced OA progression

Twelve weeks of HFD treatment induced mild changes in OA pathology in aged female mice, manifesting mainly as cartilage damage and soft tissue calcifications (Fig. 2 *A*, *B*). HFD induced significantly increased OARSI and modified‐Mankin scores in DAP12 KO mice, reaching similar scores as WT mice, indicating that DAP12 mice had accelerated OA during the 12 weeks of HFD treatment (Fig. 3 *A*, *B*). HFD treatment induced cartilage damage in DAP12 KO mice with a trend towards increased damage in WT mice (*p* = 0.0977), but no change in TLR4 KO mice (Figs. 2 *D*). No significant changes developed in tidemark duplication, synovial thickening, safranin‐O staining, or hypertrophic chondrocytes with HFD treatment independent of genotype (Fig.3 *C, E, F, G*). Two‐way ANOVA revealed significant genotype effects for the modified‐Mankin score and hypertrophic chondrocytes with trends for cartilage damage and tidemark duplication (Table 1). For diet and genotype interactions, there was a trend towards significance for cartilage damage and tidemark duplication.

**Table 1:** Two‐way ANOVA of Osteoarthritis Scores

				Fisher's LSD						
	Two‐way ANOVA			WT vs DAP12KO		WT vs TLR4KO		Control vs HF		
Measure	Genotype effect	Diet effect	Interaction	Con	HF	Con	HF	WT	DAP12 KO	TLR4 KO
Modified Mankin OA	**0.0473**	0.3356	0.1044	**0.0455**	0.2162	0.8540	**0.0122**	0.2477	0.1084	0.2331
OARSI	0.1042	0.2585	0.1041	**0.0404**	0.4777	0.8253	**0.0340**	0.3730	0.0553	0.3117
Cartilage Damage	0.0619	0.0546	0.0739	**0.0491**	0.3668	0.7984	**0.0099**	0.0977	**0.0228**	0.4679
Tidemark Duplication	0.0775	0.7515	0.0802	0.1070	0.3378	0.8756	**0.0070**	0.3842	0.2020	0.0939
Safranin‐O	0.7932	0.8203	0.4381	0.7449	0.9884	0.7928	0.2184	0.5846	0.4667	0.3553
Hypertrophic Chondrocytes	**<0.0001**	0.2720	0.9785	**<0.0001**	**<0.001**	0.1787	0.1602	0.5537	0.4610	0.5680
Synovial Thickening	0.7265	0.3887	0.8960	0.6673	0.5128	0.7470	0.9049	0.5410	0.4660	0.8895

HF = high fat.

Bold type indicates significant findings with *p* < 0.05.

**Figure 4:**
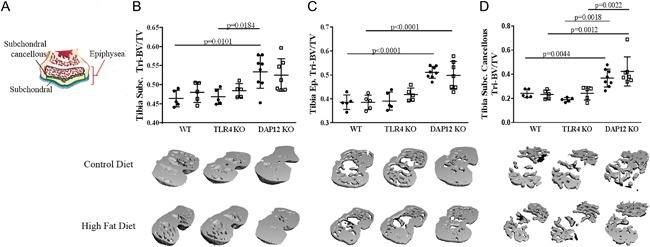
DAP12 KO mice have increased subchondral bone independent of diet. (*A*) Cartoon indicating the regions of interest for calculations of subchondral bone compartments. Black circles indicate control (CON) diet and open squares represent high‐fat (HF) diet. (*B*) Tibial subchondral bone mass (bone volume/tissue volume [BV/TV]), (*C*) tibial epiphyseal subchondral BV/TV, (*D*) tibial subchondral cancellous bone volume (BV/TV). (*B*–*D*) animal numbers WT CON (*n* = 5), WT HF (*n* = 5), TLR4 KO CON (*n* = 5), TLR4 KO HF (*n* = 5), DAP12 KO CON (*n* = 5), DAP12 KO HF (*n* = 5). Values are mean ± SD. *P* values as indicated.

MicroCT analysis revealed significantly increased total subchondral bone, tibial subchondral epiphyseal bone, and tibial subchondral cancellous bone in control‐diet DAP12 KO mice compared with WT mice (Fig. 4 *A*, *B*, *C*, *D*). No significant bone mass changes occurred in these bone compartments with 12 weeks of HFD independent of genotype.

### HFD induces significant leptin increases, but not systemic inflammation in aged female mice

**Figure 5:**
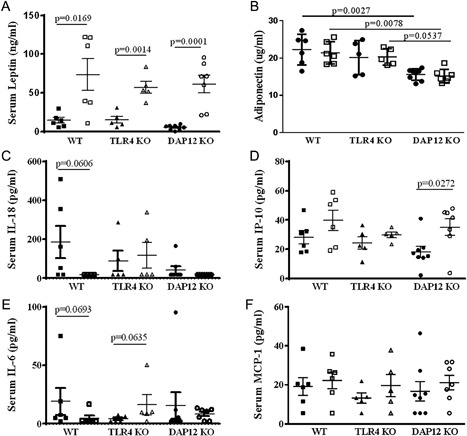
Differential effect of TLR4 and DAP12 on changes in systemic adipokines and serum cytokines in response to high‐fat diet (HFD). After 12 weeks of a control (CON) or HFD, serum adipokine leptin and serum cytokines (TNFα, IL‐1β, IL‐10, IL‐18, IL‐6, IP‐10, and MCP‐1) were measured by multiplex assay. Black circles indicate control diet and open squares represent HFD. Adiponectin was measured by ELISA. TNFα, IL‐1β, and IL‐10 were not detectable. Black symbols indicate control diet and open symbols represent HFD. (*A*) Leptin, (*B*) adiponectin, (*C*) IL‐18, (*D*) IP‐10, (*E*) IL‐6, (*F*) MCP‐1. WT CON (*n* = 5), WT HF (*n* = 6), TLR4 KO CON (*n* = 5), TLR4 KO HF (*n* = 5), DAP12 KO CON (*n* = 8), DAP12 KO HF (*n* = 7). Values are mean ± SD. *P* values are as indicated between indicated groups.

HFD induced significant elevations in serum leptin independent of genotype (Fig. 5 *A*). Serum adiponectin levels were not significantly affected by HFD in any group; however, DAP12 KO mice had significantly lower adiponectin levels compared with WT mice with control and HFD mice (Fig. 5 *B*). After 12 weeks of diet, serum was collected and analyzed by multiplex for serum cytokines, previously implicated in OA (TNFα, IL‐1β, IL‐10, IL‐18, IL‐6, IP‐10, IL‐10, and MCP‐1). TNFα, IL‐1β, and IL‐10 were undetectable in serum independent of diet or genotype in these aged females (data not shown). Serum IL‐18, IL‐10, IL‐6, and MCP1 did not differ between genotypes. Serum IL‐18, an IL‐1 family member, trended downward with HFD in WT mice (*p* = 0.0606), but not in TLR4 KO or DAP12 KO mice, likely because of already low levels (Fig. 5 *C*). Monocyte chemokine, IP‐10 (CXCL10), significantly increased in DAP12 KO mice, but not in WT and TLR4 mice (Fig. 5 *D*). IL‐6 trended downward in WT mice (*p* = 0.0693) and upward in TLR4 KO (*p* = 0.0635) mice (Fig. 5 *E*). MCP‐1 levels did not change significantly with diet or genotype (Fig. 5 *F*). These data indicate that 12 weeks of HFD treatment in aged female mice induces minimal systemic increases in proinflammatory cytokines.

### HFD induces infrapatellar fat pad inflammation and adipocyte hypertrophy

**Figure 6:**
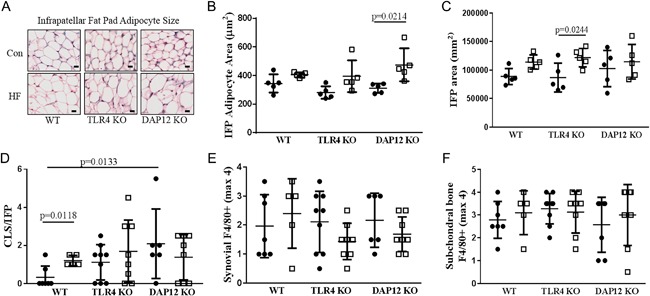
**A** high‐fat diet (HFD) induces infrapatellar fat pad adipocyte hypertrophy in the absence of TLR4 or DAP12. (*A*) Representative images of infrapatellar fat pads (IFPs) from midjoint stained with hematoxylin and eosin. Black circles indicate control (CON) diet and open squares represent HFD. (*B*) IFP adipocyte area, (*C*) total IFP area, (*D*) macrophage crown‐like structures (CLSs) per IFP section, (*E*) synovial F4/80 score (0 to 5 scale), (*F*) tibial subchondral bone marrow F4/80 score (0 to 5 scale). *N* = 5 per group for panels *B* and *C* and *N* = 5 to 9 per group for panels *D*, *E*, and *F*. Values are mean ± SD. *P* values indicate significance between groups.

Having analyzed subchondral bone and systemic mediators of OA, we investigated the IFP for histological and gene expression changes induced by HFD. HFD induced IFP adipocyte hypertrophy in DAP12 KO mice (34.3% increase), but not in WT or TLR4 KO mice (Fig. 6 *A*, *B*). After HFD treatment, the total IFP area calculated at midjoint was significantly enlarged in TLR4 KO mice and trended upward in WT mice (*p* = 0.0944) (Fig. 6 *C*). These data indicate that TLR4 and DAP12 participate in IFP adipocyte hypertrophy and fat pad enlargement in response to HFD.

We next quantified crown‐like structures (CLSs) in the IFP as a measure of chronic adipose tissue inflammation. Crown‐like structures represent dead or dying adipocytes surrounded by macrophages that are scavenging lipid droplets and are significantly increased in visceral fat with obesity.^25^ HFD significantly increased CLSs in the IFP in WT mice (Fig. 6D). Interestingly, control‐diet‐treated DAP12 KO mice had significantly more IFP CLSs and control‐diet TLR4 KO mice had a trend towards more CLSs (*p* = 0.0706) compared with control‐diet WT mice (Fig. 6 *D*). However, IFP CLSs did not change significantly with HFD in DAP12‐ or TLR4 KO mice. Synovial F4/80 staining, which is typically associated with joint inflammation secondary to macrophage infiltration, was not altered with HFD independent of genotype (Fig. 6 *E*). Moreover, F4/80 staining in subchondral bone did not differ by genotypes or diets (Fig. 6 *F*). F4/80 staining showed marked heterogeneity within groups; nevertheless, the results suggest that differences in macrophage infiltration into synovial tissue or subchondral bone were unlikely to contribute to differences in OA severity observed between genotypes.

### HFD decreases IFP and synovium inflammation and insulin signaling in DAP12 KO mice

**Figure 7:**
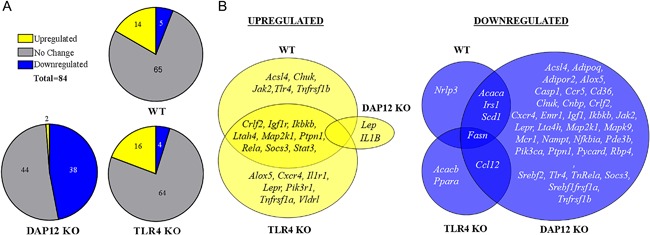
Infrapatellar fat pad and synovium gene expression is significantly downregulated with high‐fat diet in DAP12 KO mice compared with WT or TLR4 KO mice. RNA was isolated from IFP and attached synovium and analyzed using a QIAGEN RT^2^ Mouse Insulin Resistance Profiler PCR Array. (*A*) Schematic of the number of genes upregulated (yellow), downregulated (blue), or unchanged (grey) as a result of HF diet in WT, DAP12 KO, and TLR4 KO mice. (*B*) Venn diagram showing unique and overlapping HFD‐induced upregulated genes in WT, TLR4 KO, and DAP12 KO mice. (*C*) Venn diagram showing unique and overlapping HFD downregulated genes. Genes with significant changes in fold expression between HFD and control diet samples were determined based on 95% CIs using the 2^‐ddCt (HF:Control) values. Sample sizes: WT (*n* = 5), TLR4 KO (*n* = 5), and DAP12 KO (*n* = 6).

To further investigate changes in inflammation and metabolism, we used a mouse insulin‐resistance array to interrogate several pathways including insulin signaling, adipokine signaling, innate immunity, inflammation, apoptosis, and metabolism (84 genes) from the IFP with attached synovium. Genotype did not significantly alter gene expression of combined IFP and synovium samples in mice fed a control diet, with the exception of TLR4 and DAP12 genes being absent in TLR4 KO and DAP12 KO mice, respectively (data not shown). Therefore, we focused our analysis on fold‐change in gene expression induced by a HFD. Analysis revealed that a similar number of genes were upregulated and downregulated in WT and TLR4 KO mice, with about three times as many upregulated versus downregulated genes (Fig. 7 *A*, *B*). In contrast, HFD induced upregulation of only leptin and IL‐1β in DAP12 KO mice with downregulation of 38 genes (Fig. 7 *A*, *B*). Comparisons of fold‐changes between genotypes revealed significant differences between TLR4 KO and DAP12 KO mice, as well as WT and DAP12 KO mice (Table 2).

**Table 2:** Effect of Genotype on High‐Fat Diet‐Induced Changes in Synovial‐IFP Gene Expression

		Genotype comparison	HF:Control (fold‐change)					
Gene	GeneBank	(Kruskal–Wallis *p* value)	WT (*n *= 5)		TLR4 KO (*n *= 5)		DAP12 KO (*n *= 6)	
*Alox5*	NM_009662	<0.0001##	1.25 ± 0.11		2.12 ± 0.35	**	0.51 ± 0.10	
*Ikbkb*	NM_010546	<0.0001##	1.21 ± 0.06		1.64 ± 0.16	**	0.76 ± 0.07	
*Lepr*	NM_010704	<0.0001##	0.97 ± 0.14		2.04 ± 0.14	**	0.62 ± 0.08	
*Rela*	NM_009045	<0.0001##	1.30 ± 0.06		1.67 ± 0.21	**	0.78 ± 0.06	
*Crlf2*	NM_016715	0.0001##	1.39 ± 0.06		1.73 ± 0.18	**	0.79 ± 0.07	
*Lta4h*	NM_008517	0.0001##	1.15 ± 0.05		1.48 ± 0.17	**	0.63 ± 0.05	
*Map2k1*	NM_008927	0.0001##	1.25 ± 0.08		1.48 ± 0.15	**	0.74 ± 0.09	
*Pik3ca*	NM_008839	0.0002##	1.17 ± 0.06		1.41 ± 0.15	**	0.72 ± 0.07	
*Srebf2*	NM_033218	0.0002##	0.92 ± 0.10		1.73 ± 0.28	**	0.69 ± 0.04	
*Mapk9*	NM_016961	0.0003##	0.99 ± 0.09		1.44 ± 0.23	**	0.68 ± 0.03	
*Acsl4*	NM_019477	0.0004##	1.07 ± 0.11		1.74 ± 0.29	**	0.61 ± 0.09	*
*Adgre1*	NM_010130	0.0005##	1.22 ± 0.14		1.56 ± 0.30	**	0.51 ± 0.05	*
*Chuk*	NM_007700	0.0006##	1.37 ± 0.12		1.22 ± 0.21	*	0.54 ± 0.06	*
*Tnfrsf1b*	NM_011610	0.0006##	1.37 ± 0.12		1.43 ± 0.17	*	0.64 ± 0.07	*
*Socs3*	NM_007707	0.0007##	1.59 ± 0.13		1.62 ± 0.16	*	0.76 ± 0.08	*
*Ptpn1*	NM_011201	0.0014##	1.27 ± 0.05		1.64 ± 0.21	**	0.73 ± 0.06	
*Mc1r*	NM_008559	0.0015##	1.23 ± 0.14		1.45 ± 0.28	*	0.60 ± 0.09	*
*Ccl12*	NM_011331	0.0016##	1.83 ± 0.41	**	0.18 ± 0.04		0.46 ± 0.17	
*Nfkbia*	NM_010907	0.0028##	1.09 ± 0.10		1.95 ± 0.35	**	0.71 ± 0.05	
*Stat3*	NM_011486	0.0032#	1.48 ± 0.09		1.54 ± 0.17	*	0.79 ± 0.10	*
*Jak2*	NM_008413	0.0033#	1.38 ± 0.09		1.31 ± 0.18	*	0.75 ± 0.06	*
*Cxcr4*	NM_009911	0.0034#	1.30 ± 0.11		1.98 ± 0.30	**	0.65 ± 0.11	
*Pik3r1*	NM_001024955	0.0034#	1.53 ± 0.32		2.36 ± 0.42	**	0.82 ± 0.09	
*Pycard*	NM_023258	0.0034#	1.28 ± 0.16		1.49 ± 0.30	*	0.68 ± 0.09	*
*Tlr4*	NM_021297	0.0043#	1.30 ± 0.10		−		0.64 ± 0.08	**
*Il1r1*	NM_008362	0.0047#	1.33 ± 0.15		1.91 ± 0.32	*	0.74 ± 0.12	
*Ccr5*	NM_009917	0.0057#	1.27 ± 0.19		0.99 ± 0.16		0.45 ± 0.15	*
*Cnbp*	NM_013493	0.0057#	1.20 ± 0.17		1.38 ± 0.15	*	0.71 ± 0.07	
*Tnfrsf1a*	NM_011609	0.0057#	1.30 ± 0.18		1.65 ± 0.23	*	0.76 ± 0.09	
*Igf1*	NM_010512	0.0102#	1.31 ± 0.22		1.52 ± 0.37		0.63 ± 0.06	*
*Nampt*	NM_021524	0.0162#	1.29 ± 0.18		1.72 ± 0.45		0.66 ± 0.04	
*Rps6kb1*	NM_028259	0.0219	1.18 ± 0.10		1.38 ± 0.19	*	0.83 ± 0.09	
*Igf1r*	NM_010513	0.0239	1.23 ± 0.06		1.74 ± 0.12	*	1.10 ± 0.28	
*Cd36*	NM_007643	0.0252	1.26 ± 0.27		1.62 ± 0.37		0.70 ± 0.10	
*Insr*	NM_010568	0.0319	1.13 ± 0.16		1.28 ± 0.11	*	0.83 ± 0.09	
*Mtor*	NM_020009	0.0335	1.03 ± 0.04		1.48 ± 0.18	*	0.91 ± 0.10	
*Il1b*	NM_008361	0.0339	0.56 ± 0.23		0.91 ± 0.23		2.52 ± 0.64	*
*Mapk3*	NM_011952	0.0343	1.13 ± 0.12		1.54 ± 0.20	*	0.88 ± 0.13	
*Pde3b*	NM_011055	0.0370	1.54 ± 0.41		1.58 ± 0.31		0.66 ± 0.08	
*Adipor2*	NM_197985	0.0397	0.98 ± 0.22		1.17 ± 0.15	*	0.62 ± 0.07	
*Cxcr3*	NM_009910	0.0480	0.75 ± 0.12		1.62 ± 0.46		0.61 ± 0.16	
*Irs1*	NM_010570	0.055	0.81 ± 0.07		1.30 ± 0.22		0.72 ± 0.06	
*Srebf1*	NM_011480	0.092	1.09 ± 0.23		1.20 ± 0.15		0.75 ± 0.07	
*Vldlr*	NM_013703	0.103	1.36 ± 0.44		1.62 ± 0.21		0.83 ± 0.20	
*Adipoq*	NM_009605	0.113	1.37 ± 0.58		1.70 ± 0.42		0.66 ± 0.07	
*Acaca*	NM_133360	0.123	0.42 ± 0.14		0.62 ± 0.15		0.24 ± 0.02	
*Apoe*	NM_009696	0.129	0.93 ± 0.14		1.47 ± 0.36		0.76 ± 0.13	
*Ppara*	NM_011144	0.143	1.34 ± 0.26		0.80 ± 0.05		1.42 ± 0.32	
*Adipor1*	NM_028320	0.152	1.20 ± 0.15		1.26 ± 0.18		0.80 ± 0.13	
*Casp1*	NM_009807	0.173	0.96 ± 0.18		1.14 ± 0.28		0.63 ± 0.13	
*Tnf*	NM_013693	0.173	0.70 ± 0.19		0.59 ± 0.16		1.69 ± 0.64	
*Ccr6*	NM_009835	0.219	2.56 ± 1.33		6.40 ± 3.91		0.76 ± 0.24	
*Il23r*	NM_144548	0.226	1.66 ± 0.41		4.45 ± 1.67		1.73 ± 0.37	
*Scd1*	NM_009127	0.235	0.61 ± 0.10		0.65 ± 0.16		0.40 ± 0.09	
*Trem2*	NM_001272078	0.235	1.46 ± 0.21		1.55 ± 0.35		1.10 ± 0.29	
*Olr1*	NM_138648	0.311	1.27 ± 0.23		0.87 ± 0.22		0.88 ± 0.21	
*Pck1*	NM_011044	0.329	1.53 ± 0.50		1.10 ± 0.26		0.80 ± 0.08	
*Irs2*	NM_001081212	0.337	1.37 ± 0.17		1.30 ± 0.13		1.27 ± 0.27	
*Rbp4*	NM_011255	0.343	0.65 ± 0.20		0.84 ± 0.19		0.47 ± 0.14	
*Cebpa*	NM_007678	0.350	1.28 ± 0.28		1.60 ± 0.29		1.00 ± 0.13	
*Acacb*	NM_133904	0.380	0.67 ± 0.19		0.43 ± 0.12		1.16 ± 0.37	
*Lep*	NM_008493	0.383	2.85 ± 1.03		5.14 ± 1.63		2.08 ± 0.29	
*Cs*	NM_026444	0.394	1.22 ± 0.33		1.14 ± 0.11		0.90 ± 0.35	
*Serpine1*	NM_008871	0.408	1.46 ± 0.31		1.90 ± 0.54		0.96 ± 0.14	
*Pparg*	NM_011146	0.416	1.11 ± 0.30		1.45 ± 0.26		0.86 ± 0.13	
*Fabp4*	NM_024406	0.423	1.20 ± 0.27		1.28 ± 0.37		0.87 ± 0.15	
*Lpl*	NM_008509	0.423	1.13 ± 0.25		1.25 ± 0.27		0.79 ± 0.11	
*Cd3e*	NM_007648	0.427	0.72 ± 0.22		1.29 ± 0.33		0.84 ± 0.22	
*Akt3*	NM_011785	0.447	1.28 ± 0.10		1.71 ± 0.34		2.05 ± 0.47	
*Il18r1*	NM_008365	0.478	1.34 ± 0.41		1.65 ± 0.45		0.99 ± 0.36	
*Retn*	NM_022984	0.478	1.15 ± 0.35		0.90 ± 0.08		1.41 ± 0.35	
*Lipe*	NM_010719	0.482	1.24 ± 0.41		1.79 ± 0.31		1.22 ± 0.23	
*Pdk2*	NM_133667	0.520	1.99 ± 0.72		0.86 ± 0.17		1.85 ± 0.65	
*Slc2a4*	NM_009204	0.520	0.92 ± 0.17		1.20 ± 0.10		1.22 ± 0.19	
*Fasn*	NM_007988	0.548	0.29 ± 0.11		0.29 ± 0.05		0.35 ± 0.19	
*Slc27a1*	NM_011977	0.601	0.92 ± 0.16		1.15 ± 0.14		0.96 ± 0.24	
*Ppargc1a*	NM_008904	0.656	1.08 ± 0.19		1.38 ± 0.19		1.27 ± 0.30	
*Ucp1*	NM_009463	0.697	0.68 ± 0.28		1.93 ± 0.81		0.75 ± 0.43	
*Nlrp3*	NM_145827	0.712	0.65 ± 0.09		1.04 ± 0.30		0.79 ± 0.24	
*Acsl1*	NM_007981	0.773	1.09 ± 0.30		0.83 ± 0.16		0.76 ± 0.12	
*Tyrobp*	NM_011662	0.802	1.19 ± 0.12		1.24 ± 0.21		‐	
*Itgax*	NM_021334	0.845	1.38 ± 0.30		1.22 ± 0.29		1.34 ± 0.34	
*Gys1*	NM_030678	0.904	1.21 ± 0.30		1.00 ± 0.21		1.40 ± 0.38	
*Hk2*	NM_013820	0.924	1.25 ± 0.26		1.10 ± 0.11		1.19 ± 0.32	

HF = high fat.

Fold‐change values are mean ± SEM. ##*p *< 0.01 and #*p *< 0.05 following Benjamini–Hochberg adjustment for multiple comparisons. **p < 0.01 and *p < 0.05 for comparisons between adjacent genotypes using Dunn's multiple comparison post hoc test. Final column compares DAP12 and WT mice.

**Figure 8:**
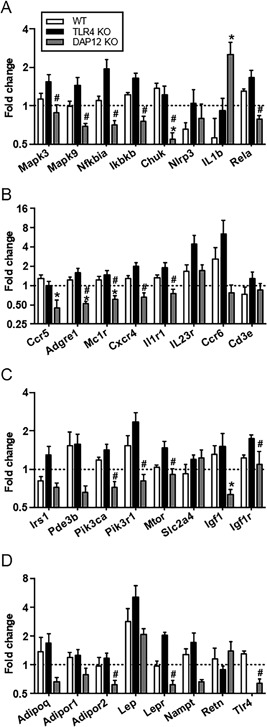
High‐fat diet (HFD) reduces the expression of inhibitors of inflammatory signaling, adipokines, and insulin signaling in infrapatellar fat pad and synovial tissue from DAP12 KO mice. HFD‐induced fold‐change in expression of (*A*) inflammatory signaling genes downstream of DAP12 or TLR4, (*B*) leukocyte infiltrating markers, (*C*) genes involved in insulin signaling, and (*D*) adipokine signaling pathways. The fold‐change expression between high fat and control diet samples was determined based on 2^‐ddCt (HF:Contol). *Y*‐axis in Log2 scale. Sample sizes: WT (*n* = 5), TLR4 KO (*n* = 5), and DAP12 KO (*n* = 6). *p < 0.05 between DAP12 KO and WT mice; #*p* < 0.05 between DAP12 KO and TLR4 KO mice.

Further analysis revealed opposing roles of TLR4 and DAP12 in several genes involved in inflammatory signaling (Fig. 8 *A*). Negative regulators of NF‐κB signaling (*Nfkbia, Ikbkb,* and *Chuk*) were suppressed in DAP12 KO IFP accompanied by increased procartilage catabolism factor IL‐1β (Fig. 8 *A*). Leukocyte infiltration markers (*Erm1, Ccr5, Cxcr4,* and *Crlf2*) were also downregulated in DAP12 KO mice (Fig. 8 *B*). Despite elevated systemic insulin levels in DAP12 KO mice, the expression of local insulin signaling mediators (*InsR, Igf1, Pi3kca,* and *Pik3r1*) within the IFP were suppressed with HFD treatment in the absence of DAP12 (Fig. 8 *C*). With the exception of leptin and resistin, which were similar between genotypes, adipokine signaling (*Adipor2, Lepr, Socs3,* and *Stat3*) was also suppressed in DAP12 KO mice compared with WT or TLR4 KO mice (Fig. 8 *D*). Interestingly, the expression of TLR4 and nicotinamide phosphoribosyl‐transferase (*Nampt*), an adipokine ligand that binds and activates TLR4, was reduced in HFD‐treated DAP12 KO mice compared with HFD‐treated WT mice (Fig. 8 *D*).^26^ The expression of genes involved in fatty acid metabolism (*Alox5, Lta4H, Cd36, Acls4, Rpsbkb1,* and *Mtor*) was also suppressed in DAP12 KO mice compared with TLR4 KO mice (Table 2). These data highlight the significant differences in IFP and synovium gene expression induced by HFD in the absence of TLR4 or DAP12. Overall, TLR4 and DAP12 have opposing roles in regulating the expression of genes involved in insulin and adipokine signaling, inflammation, and fatty acid metabolism within the IFP.

## Discussion

Although obesity increases the incidence of knee OA in a female sex and age‐dependent manner in people,^3^ most preclinical animal models of diet‐induced obesity and OA have initiated HFD in weanling or young male mice.^27^ Our study is unique in several ways. First, we chose to use aged female mice based on the highest prevalence of obesity and OA progression in middle‐aged and elderly women.^28^ Second, we chose to use a short feeding strategy of only 12 weeks to capture early factors induced by a very HFD that would accelerate age‐associated OA. Third, we evaluated the contribution of innate immune TLR4 signaling in HFD‐induced OA. Our findings support an essential role for TLR4 in the progression of HFD‐induced knee OA. Despite becoming obese and glucose intolerant, aged female TLR4 KO mice treated with HFD were protected against articular cartilage damage and tidemark duplication, resulting in significantly reduced OARSI scores compared with HFD‐treated WT mice. Meanwhile, DAP12 KO mice, unable to inhibit TLR4 cytokine responses, developed accelerated cartilage catabolism on a HFD. These data indicate that HFD‐induced OA are modulated by strength of TLR4 signaling.

Based on prior studies in young male TLR4 KO mice,^10^, ^13^, ^14^, ^15^ we hypothesized that TLR4 KO mice would be protected from obesity‐associated inflammation and insulin resistance leading to protection from HFD‐induced knee OA progression. We found that aged female TLR4 KO mice treated with HFD for 12 weeks developed significant obesity and glucose intolerance, although not as severe as WT mice, with associated hyperinsulinemia. We also discovered that DAP12 KO mice had hyperinsulinemia independent of diet and had improved systemic glucose tolerance. Recently, insulin was shown to inhibit TNFα‐induced OA‐related catabolic genes in fibroblast‐like synoviocytes from OA joints; therefore, it is possible that hyperinsulinemia seen in control‐diet DAP12 KO mice may provide some aged‐related OA protection that we observed.^29^ Thus, changes in glucose homeostasis alone do not explain the protection from HFD‐induced OA in TLR4 KO mice and the acceleration of HFD‐induced cartilage catabolism seen in DAP12 KO aged female mice.

Adipokines provide another link between adiposity and OA. Leptin induces macrophage activation, potentiates TLR responses, and promotes OA as evidenced by OA protection in the extremely obese leptin‐ or leptin‐receptor‐deficient mice.^30^, ^31^ However, we did not find any significant differences in serum leptin levels between genotypes with HFD treatment. Within the synovium and IFP, leptin and leptin receptors were significantly upregulated in TLR4 KO tissue; whereas DAP12 KO tissue had significantly reduced leptin receptors coupled with increased leptin. More studies are needed to determine whether the strength of leptin signaling is mediating OA pathology in this model.

Adiponectin, an anti‐inflammatory adipokine, is significantly downregulated by obesity and inversely correlated with OA progression.^(9)^ In our model, adiponectin levels did not change significantly with diet despite a doubling of percent body fat with HFD. WT and TLR4 KO mice had similar levels of adiponectin, but DAP12 KO mice had very low levels independent of diet. Given this inverse relationship, we would have predicted DAP12 KO mice would have worsened instead of experiencing reduced age‐related OA. Adiponectin does have insulin‐sensitization properties; it is possible that low adiponectin in DAP12 KO mice contributes to reduced insulin signaling seen in the IFP.

The evaluation of HFD‐induced changes in the IFP revealed some significant differences between WT, TLR4 KO, and DAP12 KO mice. IFP adipocytes became hypertrophic in DAP12 KO mice, but not in WT or TLR4 KO mice. Hypertrophy of adipocytes indicates an improved ability to store dietary fatty acids as triglycerides, and is often accompanied by increased expression of fatty acid transporters including CD36. However, HFD significantly downregulated *CD36*, as well as other genes involved in fatty acid metabolism (*Acls4*, *Cnep*, *Srebf2*, and *LepR*) in DAP12 KO IFP, suggesting locally dysregulated fatty acid metabolism. In addition to adipocyte hypertrophy, the relative increase in IFP total area was most significant in TLR4 KO mice. Clinically, increased IFP size has been associated with protection against knee OA in women, through an unknown mechanism,^32^ but inflammation suppresses IFP adipogenesis^33^ and may contribute to the negative association between IFP size and knee OA progression in humans. However, with regard to adipose tissue inflammation, we found similar numbers of crown‐like structures in the IFP of HFD mice independent of genotype, suggesting that local IFP macrophage infiltration was not the determinant for HFD‐induced OA progression.

We observed that HFD increased TLR4 expression in the IFP and synovium of WT mice, consistent with obesity‐induced upregulation of TLR4‐dependent signaling in joint tissues. Transcriptionally, IFP from WT and TLR4 KO mice had very similar patterns of gene upregulation in areas of lipid metabolism and signaling (eg, *Lepr, Alox5*, and *Vldrl*), insulin signaling (*Pik3r1*), and the regulation of inflammation (eg, *Ilr1, Tnfrsf1a*, and *Cxcr4*). On the other hand, IFP from DAP12 KO HFD mice had significant downregulation of these same pathways, as well as downregulation of inhibitors of inflammatory signaling including *Chuk*, *Nfkbai*, and *Ikbkb*, and inhibition of cytokine signaling, such as *Socs3*. Additional studies are required to determine if upregulation IL‐1β and dysregulated fatty acid metabolism or leptin signaling within the IFP/synovium of HFD‐treated DAP12 KO mice contributes to the accelerated cartilage catabolism seen in these mice.

Our study has several limitations. First, we used global TLR4‐ and DAP12‐deficient mice. A conditional KO mouse of TLR4 and DAP12 selectively deleted in chondrocytes, macrophages, or adipocytes would provide additional information regarding the role of DAP12 and TLR4 in HFD‐induced OA. Second, we only addressed aged female mice and do not know if the results are similar to male mice or younger mice. Third, we used a very HFD (60% kcal fat) that though similar in fat caloric content to some low‐carbohydrate diets (eg, the Atkin's diet) has greater fat content than a typical human diet. However, despite these limitations, these results provide new insights into the pathophysiology of HFD‐diet‐induced OA.

In conclusion, we have shown that TLR4 is required for and DAP12 protects against HFD‐induced OA progression in aged female mice. Our data indicate that TLR4 and DAP12 have specific effects on cartilage damage, IFP gene expression, and subchondral bone in this model. Future studies should focus on the TLR4‐ and DAP12‐dependent mediators of obesity‐induced cartilage catabolism.

## Disclosures

All authors report that they have no conflicts of interest.
